# The influence of covalent and non-covalent functionalization of GNP based nanofluids on its thermophysical, rheological and suspension stability properties

**DOI:** 10.1039/c9ra07811h

**Published:** 2019-11-25

**Authors:** Omar A. Hussein, Khairul Habib, R. Saidur, Ali S. Muhsan, Syed Shahabuddin, Omer A. Alawi

**Affiliations:** Department of Mechanical Engineering, Universiti Teknologi PETRONAS 32610 Bandar Seri Iskandar Perak Darul Ridzuan Malaysia khairul.habib@utp.edu.my +60 53687146; Mechanical Engineering Department, College of Engineering, Tikrit University Tikrit Iraq; Research Centre for Nano-Materials and Energy Technology (RCNMET), School of Science and Technology, Sunway University Malaysia; Department of Engineering, Lancaster University LA1 4YW UK; Petroleum Engineering Department, Universiti Teknologi PETRONAS 32610 Bandar Seri Iskandar Perak Malaysia; Department of Thermofluids, School of Mechanical Engineering, Universiti Teknologi Malaysia 81310 UTM Skudai Johor Bahru Malaysia

## Abstract

Covalent functionalization (CF-GNPs) and non-covalent functionalization (NCF-GNPs) approaches were applied to prepare graphene nanoplatelets (GNPs). The impact of using four surfactants (SDS, CTAB, Tween-80, and Triton X-100) was studied with four test times (15, 30, 60, and 90 min) and four weight concentrations. The stable thermal conductivity and viscosity were measured as a function of temperature. Fourier transform infrared spectroscopy (FTIR), thermo-gravimetric analysis (TGA), X-ray diffraction (XRD) and Raman spectroscopy verified the fundamental efficient and stable CF. Several techniques, such as dispersion of particle size, FESEM, FETEM, EDX, zeta potential, and UV-vis spectrophotometry, were employed to characterize both the dispersion stability and morphology of functionalized materials. At ultrasonic test time, the highest stability of nanofluids was achieved at 60 min. As a result, the thermal conductivity displayed by CF-GNPs was higher than NCF-GNPs and distilled water. In conclusion, the improvement in thermal conductivity and stability displayed by CF-GNPs was higher than those of NCF-GNPs, while the lowest viscosity was 8% higher than distilled water, and the best thermal conductivity improvement was recorded at 29.2%.

## Abstract 2

Covalent functionalization (CF-GNPs) and non-covalent functionalization (NCF-GNPs) approaches were applied to prepare graphene nanoplatelets (GNPs).

## Graphical abstract

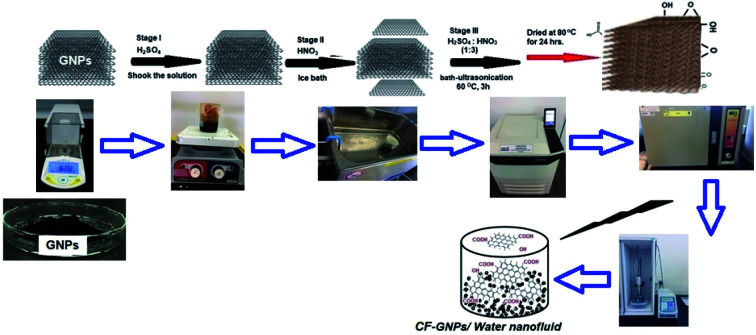

## 1. Introduction

Nanofluid refers to the stable and homogenous suspension exhibited by nanoparticles (NPs) within conventional working fluids (*e.g.*, ethylene glycol, oil, and water).^1,2^ Prior studies reported that the addition of a small weight concentration of NPs could improve the efficiency of heat transfer and thermal conductivity in the base fluid, and hence the suitability for wide range applications, such as in solar collectors, cooling appliances, and heat exchangers.^3,4^ Nanoparticles (NPs) are made of either metal oxide (*e.g.*, Al_2_O_3_, SiO_2_, CuO, and TiO_2_)^5–7^ or carbon-based particles (*e.g.*, carbon nanotube (CNT)),^8,9^ graphene oxide (GO),^10^ or graphene nanoplatelets (GNPs).^11,12^ Numerous types of NPs have been proposed in the last decades to generate nanofluids, wherein CuO, Al_2_O_3_, ZnO, and SiO_2_ are more commonly employed than nanomaterials based on metal oxide.^13–15^ Both graphene and carbon-based NPs (*e.g.*, CNT)^16^ have been assessed empirically.^12,17–20^ Simply put, nanofluids have garnered much attention due to their chemical and physical properties, as well as their nanometre size. More importantly, nanofluids have improved thermal conductivity, hence their suitability to serve as heat-exchanging fluids. Upon inclusion of a NP base fluid, some empirical studies have also improved the convective heat transfer, in comparison to the pure base fluid.

Choi^1^ discovered an improvement in thermal conductivity after including copper NPs into the base fluids. Meanwhile, Zeinali Heris *et al.*,^21^ assessed the convective heat transfer coefficient of CuO NPs, wherein the outputs were compared with Al_2_O_3_–water nanofluid. The reported outcomes displayed a higher convective heat transfer coefficient for Al_2_O_3_–water nanofluids than CuO–water nanofluids. Here, the integral role of NPs in thermal conductivity is highlighted for nanofluid heat transfer. A maximum decrease of 24% was noted for thermal resistance in water-based titanium dioxide and gold nanofluids.^22^ Shanbedi *et al.*,^23^ determined the impact of multi-walled carbon nanotubes (MWCNT) on the effectiveness of two-phase closed thermosyphon (TPCT). As a result, 11% improvement was recorded for thermal efficiency at 90 kW in TPCT with functionalized MWCNT. Thermo-physical properties, such as thermal conductivity of NPs, have a significant function in heat transfer appliances.^24,25^ The thermal conductivity of GNPs exceeded that displayed by other carbon allotropes, such as diamond, MWCNTs, and SWCNT.^26^ The GNPs have vast applications due to its exceptional properties, particularly in the scientific domain, for instance in making batteries and sensors.^27^ Nevertheless, most applications are from being realized due to the weak interaction GNPs has with other materials. Hence, to enhance this interaction, covalent (amino acids) and non-covalent (NC) functionalization have been proposed as the solution in other studies.^28^

Covalent and non-covalent functionalization can improve the aspect of the dispersibility of GNPs in organic/aqueous solvents. The NCF of carbon nanostructures may be executed by using a range of surfactants.^28–30^ Four surfactants can be applied to enhance carbon nanostructure dispersibility in an aqueous medium, namely Tween-80 (Tw-80), sodium dodecyl sulphonate (SDS), Triton X-100 (Tx-100), and Cetyltrimethylammonium bromide (CTAB). The benzene function in Tx-100 generates powerful π–π interaction with the surface of carbon nanostructures. Besides, Tw-80 possesses better dispersibility than Tx-100, mainly due to the hindrance of steric in tip chains of Tx-100, thus the low concentration in Triton on the surface of carbon nanostructures.^31^ Despite the exceptional dispersion of carbon nanostructures in Tx-100, when compared to CTAB and SDS, numerous issues emerge due to increased mixture viscosity, such as drop in pressure in thermal appliances.^23^ Hence, Tw-80 was chosen in this study to synthesise NCF nanofluid. The CF is composed of hydrophilic or hydrophobic groups in light of high-energy characteristics, for example, GNPs edges.

In this study, GNPs were functionalized using two approaches, namely covalent and non-covalent reactions, for comparison purposes. The experiment included rapid NCF of GNPs-surfactant and CF with carboxyl groups. Next, characterization of samples and performance of thermal were assessed. The effects of CF and NCF on thermal conductivity were investigated, while the aspect of viscosity was assessed by considering a range of concentrations and temperatures. The significant increment of the viscosity of suspension vitiates in the NCF groups improved the feature of heat transfer in GNPs nanofluids. The outputs revealed the more improved thermo-physical property in covalent nanofluids (CF-GNPs) than those of water and non-covalent nanofluids (GNPs-surfactant).

## 2. Materials and methods

### 2.1. Nanoparticles and chemicals

GNPs with the purity of 98%, the maximum particle diameter of 2 μm and specific surface area 750 m^2^ g^−1^ purchased from, XG Sciences, Lansing, MI, USA, were used in this study. Sulfuric acid 95–97% (H_2_SO_4_) and nitric acid 65% analytical reagent grade (HNO_3_) were purchased from Sigma-Aldrich Co., Selangor, Malaysia, were used as the functionalization media. All chemicals were used as received without further purification. Surfactants used were SDS, CTAB, Tween-80 and Tx-100 which are purchased from Sigma-Aldrich Co., Selangor, Malaysia.

### 2.2. Covalent functionalization method

**Fig. 1:**
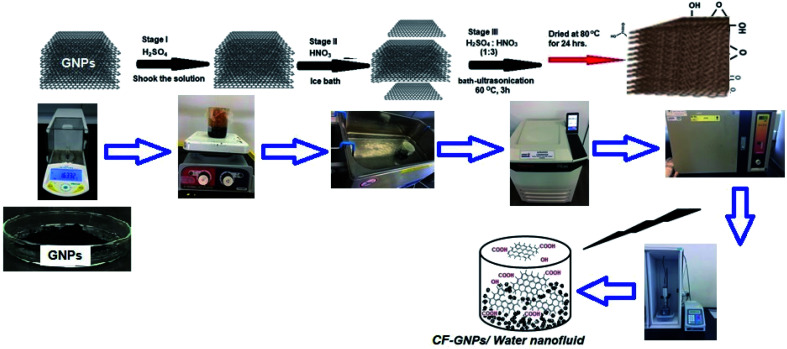
Schematic of functionalization and preparation process nanofluids.

Meanwhile, GNPs are obviously hydrophobic and it cannot be dispersed in any solvent which is polar like distilled water. Proper ways to make GNPs hydrophilic do functionalization by acid treatment. This functionalization procedure helps to present functional groups such as carboxyl and hydroxyl groups on the surface of GNPs. The pristine GNPs was transferred into H_2_SO_4_ and the solution was shaking, next, the container was moved to an ice bath and the nitric acid drops were added to the solution mixture. The ratio of sulfuric to nitric (3 : 1) was used due to its preference as an acid treatment^32,33^ then the solution was stirred for 30 min at room temperature and then was under bath-ultrasonication for 3 h. After 3 h, of the probe sonication process, the sample mixture was refluxed for 30 min at room temperature with constant stirring. After that GNPs were washed a number of times with distilled water thoroughly then centrifuged at 6000 rpm for 15 min to remove excess acid and then dried under drying oven for 24 h at 80 °C. Rich samples of (0.02, 0.05, 0.08, and, 0.1 wt%) were prepared with the addition of a known amount of functionalized GNPs into distilled water. Fig. 1 presented the schematic diagram of the functionalization and CF-GNPs preparation process of the nanofluids.

### 2.3. Non-covalent functionalization method

**Fig. 2:**
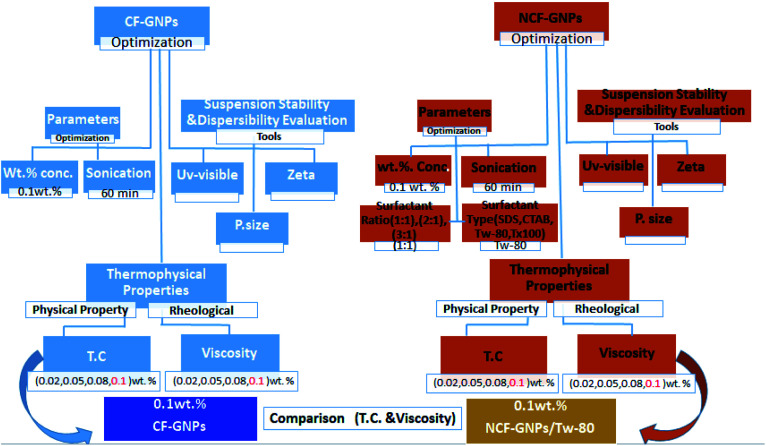
Flowchart for preparation and optimization nanofluids.

Ultrasonication probe sonication was used to dispersed GNPs in distilled water (Sonics Vibra-Cell, VC 750, Sonics & Materials, Inc., USA) having an output power of (750 W) and a power source of (20 kHz) frequency. The lack of a surfactant or functionalization cannot be stably dispersed carbon-based nanoparticles in water, ever since they have a hydrophobic surface.^34^ Accordingly, NCF-GNPs were achieved using different additives surfactants; SDS, CTAB, Tween-80 and Triton X-100. Four different ultrasonication times of 15, 30, 60 and 90 min were used. Weight concentrations of GNPs were in the range of 0.02, 0.05, 0.08, and, 0.1 wt% for the preparation of samples. Samples compositions are presented in Table 1 and Fig. 2. The NCF-GNPs weight concentration ratio (1 : 1) and 60 min GNPs were reserved constant during the preparation of samples. During the probe sonication process caused a considerable increase in heat generated in the temperature and evaporation in water of the sample with the subsequent alteration in the weight concentration of the sample. Therefore, ice bath used to regularly the temperature at a suitable degree. For long periods and stable suspension without sedimentation the prepared nanofluid would be an agglomerate-free. The weight percentage of GNPs was calculated based on eqn (1).^35^1
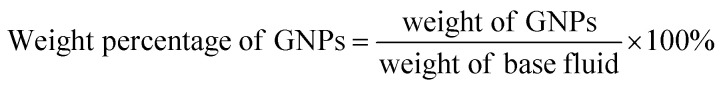


**Table 1:** Details of the samples prepared in this research with different concentrations after preparation

Sample no.	Ultrasonication time (min)	Surfactant type	Surfactant-GNPs ratio	Weight concentration (wt%)
1	30	P-GNPs	—	0.02
2	60	P-GNPs	—	0.02
3	90	P-GNPs	—	0.02
4	15	SDS	1 : 1	0.02
5	30	SDS	1 : 1	0.02
6	60	SDS	1 : 1	0.02
7	60	CF-GNPs	—	0.02
8	60	CF-GNPs	—	0.05
9	60	CF-GNPs	—	0.08
10	60	CF-GNPs	—	0.1
11	60	SDS	0.5 : 1	0.02
12	60	SDS	1 : 1	0.02
13	60	SDS	1.5 : 1	0.02
14	60	SDS	1 : 1	0.02
15	60	SDS	1 : 1	0.05
16	60	SDS	1 : 1	0.08
17	60	SDS	1 : 1	0.1
18	60	CTAB	1 : 1	0.02
19	60	CTAB	1 : 1	0.05
20	60	CTAB	1 : 1	0.08
21	60	CTAB	1 : 1	0.1
22	60	Tw-80	1 : 1	0.02
23	60	Tw-80	1 : 1	0.05
24	60	Tw-80	1 : 1	0.08
25	60	Tw-80	1 : 1	0.1
26	60	Tx-100	1 : 1	0.02
27	60	Tx-100	1 : 1	0.05
28	60	Tx-100	1 : 1	0.08
29	60	Tx-100	1 : 1	0.1

### 2.4. Measurement devices

#### 2.4.1. Evaluation of stability

Zeta potential and ultraviolet-visible spectrophotometry (UV-vis) analysis were conducted to assess the stability of GNPs dispersions. The measurement of light absorbance of a suspension by UV-vis spectroscopy can be used to make available a quantifiable characterization of the stability. The use of UV-vis is Lambda operating in the range of 200–800 nm wavelengths (UV-800/900, Lambda Company, U.S.A.). Light absorbance was measured by special quartz cuvettes suitable for the UV region since all samples were at certain time intervals for an extra 30 days. A 1 : 20 ratio diluted in distilled water to allow proper light transmission for all samples. Zetasizer Nano ZS (Anton Paar, GmbH Ltd, Malvern, UK) measured zeta potential using the prepared nanofluids principle of Electrophoretic Light Scattering (ELS). The degree of revulsion in the measurement of zeta potential between close particles shows the same load in nanofluid dispersal.^36^

#### 2.4.2. Morphology and elemental analysis

Using Fourier transform infrared (FTIR) spectroscopy, Raman spectroscopy, Field Emission Transmission Electron Microscope (FETEM, HT 7700, Hitachi) and Field Emission Scanning Electron Microscopy (FESEM, SU8000, Hitachi), the main structure of CF-GNPs nanofluids was analyzed. The evaluation of the FTIR spectra within a wavenumber range of 400–4000 cm^−1^ for FTIR spectroscopy. Phase compositions were determined by using an X-ray diffractometer (XRD, EMPYREAN, PANALYTICAL) with Cu-Kα radiation over a 2θ range from 20° to 80°. The “PANalytical X'Pert HighScore” software was employed to compare the XRD profiles with the standards compiled by the Joint Committee on Powder Diffraction and Standards (JCPDS).

#### 2.4.3. Measurement of thermophysical properties

To measure the steady-shear rheological properties of water and water-based GNPs nanofluids, Anton Paar rotational rheometer (Model Physica MCR 301, Anton Paar GmbH Ltd, Malvern, UK) was used. The temperatures in the ranges of (20–60 °C) at a shear rates of 200 s^−1^ were used to achieve the tests for all samples. Thermal conductivity was determined by the thermal property's analyzer device KD2 Pro (Decagon Devices, Inc., USA), with an accuracy of approximately 5%. A 1.4 kW water bath WNB22 Memmert (Germany) was used to keep the samples at the preferred temperature during measurements and 0.1 °C accuracy. The rotational rheometer consists of a moving cylindrical plate and a stationary cylindrical surface which are parallel with a small gap between them.

## 3. Results and discussion

### 3.1. Characterization of CF-GNPs nanoparticles

**Fig. 3:**
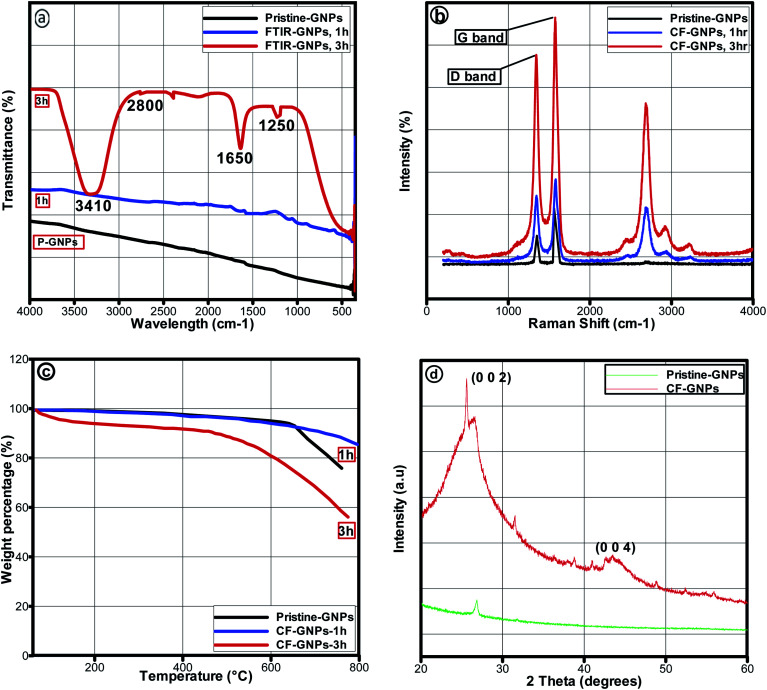
(a) FTIR spectra of P-GNPs and 1 and 3 h CF-GNPs, (b) Raman spectra of P-GNPs and 1 h, 3 h CF-GNPs, and (c) TGA curves of P-GNPs, 1 and 3 h CF-GNPs, (d) XRD pattern of CF-GNPs.

The FTIR spectra of pristine-GNPs and CF-GNPs are illustrated in Fig. 3(a). Table 2 summarises the assigned bonds and IR peaks derived from the CF-GNPs FTIR spectra. Fig. 3(a) displays the absorbance peaks of CF-GNPs at 2800, 1650, and 1250 cm^−1^, which are attributed to C–H, C=O, and C–O bonds, respectively. The bonds reflect the attachment of acid treatment molecules on the surface and edge of GNP sheets. Both bonds of C=O and C–O signify the successful direct esterification reaction. The CF-GNPs showed a broad absorbance peak at wavenumbers between 3000 and 3500 cm^−1^; attributable to –OH chains from acid treatment.^37,38^ The CF-GNPs peaks indicate the success of the CF procedure.

**Table 2:** Interpretation of the IR peak for CF-GNPs

Wavenumber at which peak occurs (cm^−1^)	Interpretation
3410	–OH stretching vibration
3000–2800	C–H stretching vibration
1650	C=O stretching vibration of carboxylic group
1250	C–O stretching vibration

The Raman analysis had been performed to determine the success of the covalent approach. In this analysis, G-band displays the existence of sp^2^ carbon, D-band shows the defects of chemical construction.^39^Fig. 3(b) illustrates the Raman spectra of P-GNPs and CF-GNPs. Ratios *I*_D_/*I*_G_ of P-GNPs and CF-GNPs are 0.843 and 0.855, respectively (see Table 3). Ratio *I*_D_/*I*_G_ for 3 h was higher for CF-GNPs than P-GNPs because of the existence of covalent new bonds on carbon sheets deriving from CF-GNPs.

**Table 3:** Displacement and intensity ratios for P-GNPs and CF-GNPs obtained from Raman spectroscopy

Sample ID	D-Band (cm^−1^)	G-Band (cm^−1^)	*I*_D_/*I*_G_ ratio
Pristine GNPs	1330	1577	0.843
CF-GNPs 1 h	1345	1584	0.849
CF-GNPs 3 h	1356	1587	0.855

Fig. 3(c) illustrates the TGA curve of P-GNPs, as well as 1 and 3 h CF-GNPs. The initial loss of weight reflected in pristine GNPs and CF-GNPs were 7% and 10%, respectively, at 0–100 °C, attributable to adsorbed moisture. Slight loss of weight was noted at 60–800 °C range for P-GNPs; attributable to the pyrolysis of primary carbon-based structures.^33^ On the contrary, the second slight weight loss by 16% was recorded for 100–500 °C range for CF-GNPs, mainly because of the formation of hydroxyl and carboxyl groups in oxidation step as unstable organic moieties, as well as degradation in the functionality of acid treatment.^38^ The third slight weight loss by 24% was recorded for 500–800 °C range for FC-GNPs, mainly because of the degradation of the graphitic structures in air (see Table 4).

**Table 4:** Mass loss composition in GNPs

Weight loss		
Sample (°C)	Pristine-GNPs (%)	CF-GNPs (%)
100	7	10
100–500	8	16
500–800	14	24

The energy dispersive X-ray (XRD) test was conducted to find the purity and degree of oxidization of the material. Fig. 3(d) shows the XRD patterns of functionalized GNPs. Two clear distinct peaks of plane (002) at around 25° and the plane (004) at 44.4° represent the structure of GNPs. The results show that the CF-GNPs did not affect the crystalline structure of GNPs which conforms with the results obtained by Yarmand *et al.*^40^

**Fig. 4:**
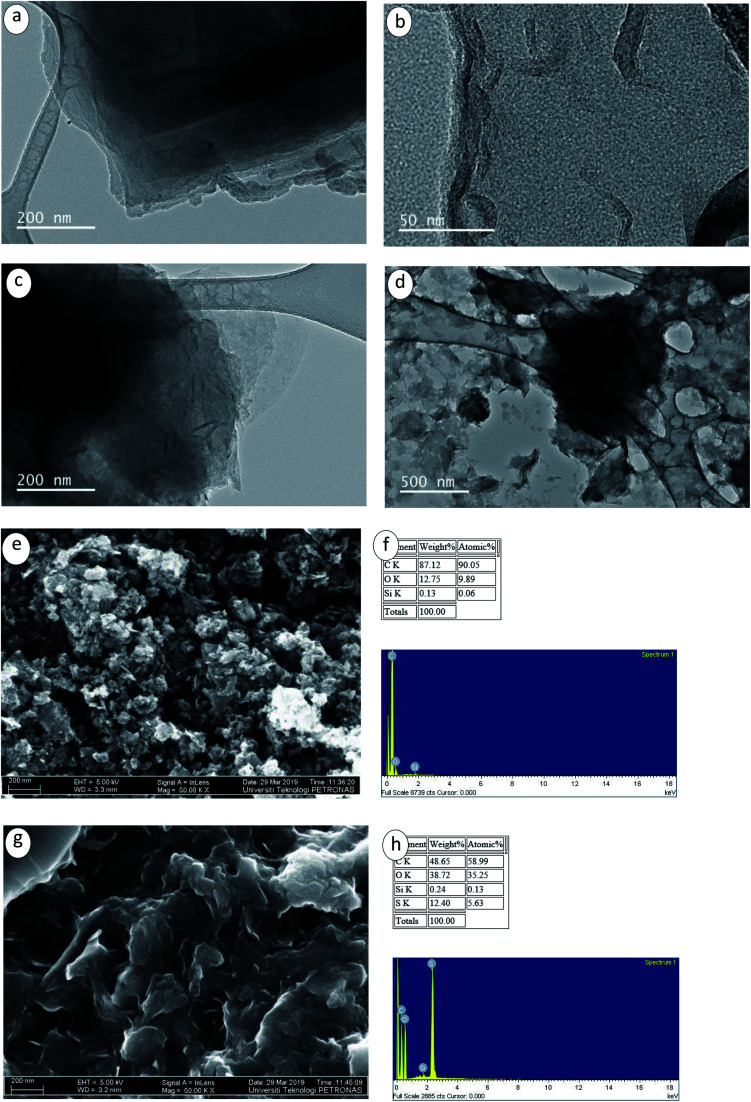
(a) FETEM images of P-GNPs and (b–d) CF-GNPs at different magnifications. FESEM and EDX images of (e and f) P-GNPs and (g and h) CF-GNPs.

The morphologies of pristine GNPs and CF-GNPs *via* FETEM and FESEM are portrayed in Fig. 4. Based on Fig. 4(a), pristine GNPs was composed of dual sheets with intact edges, transparent structures, and smooth surfaces. After functionalization, acid treatment, as well as carboxyl group on the edges and surfaces of GNPs, gave slightly blur effect on the sheets with wrinkles and crumples (see Fig. 4(b) and (c)). Changes in morphology and functional groups analysis point out the successful reaction between acid molecules and GNP-COOH. Defective folded flakes and rough edges (see Fig. 4(d)) signify the success of the CF method. FESEM images also confirm that the functionalization of GNPs results in the broken sheets of GNPs (see Fig. 4(e) and (g)). The EDX measurements portray two components present in CF-GNPs; carbon and oxygen. Despite the best dispersion stability offered by the small sheets, the thermal conductivity may be reduced slightly.^41^ On the contrary, the functionalization treatment of nanofluids explains the upper surface roughness found in P-GNPs. Damage noted partially in graphitic carbon implies higher roughness, in effect of basic carboxylation. Lines observed in FETEM images refer to wrinkles on GNPs surface as a result of inherent stability in 2D structures. The functionalization method increases in these lines during sonication, which could be due to higher than prior wrinkling or waviness.

**Fig. 5:**
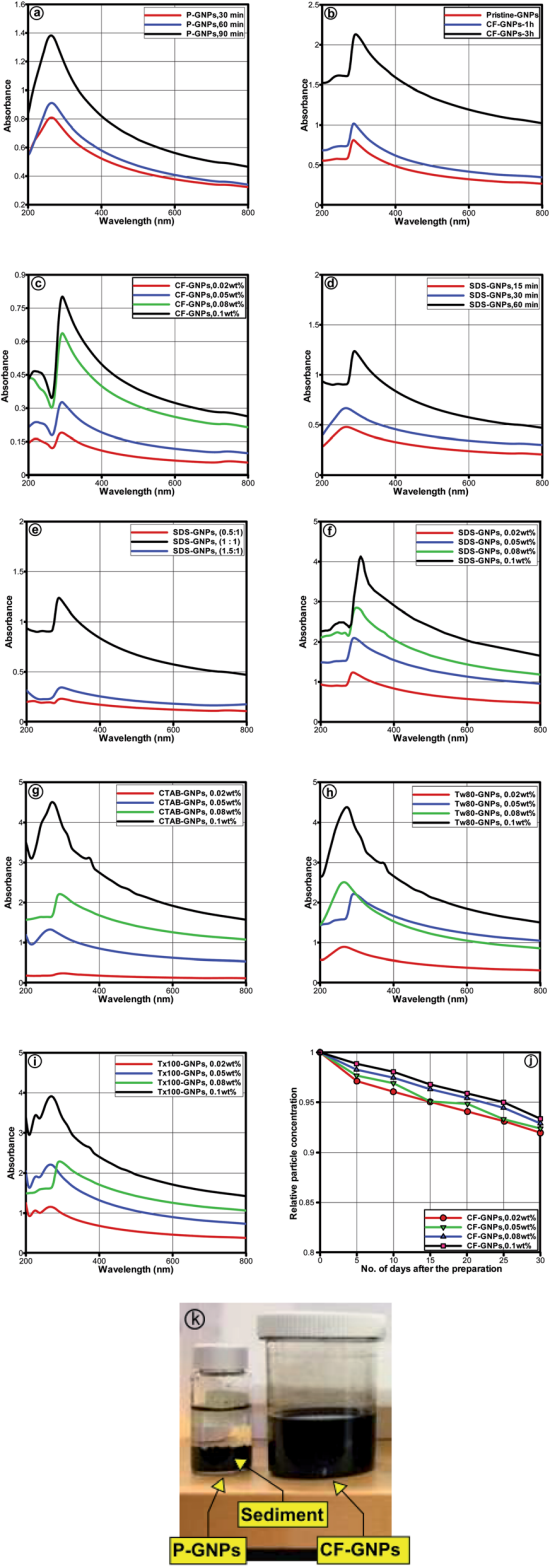
UV-vis absorption spectra of CF-GNPs nanofluids and different surfactant (a–c) P-GNPs and CF-GNPs solutions (d and e) SDS-GNPs nanofluids at different ultrasonication probe times, and different surfactant ratio (f–i) GNPs nanofluids with different particle concentrations, different surfactant and 60 min ultrasonication probe time. (j) Variation of the relative concentration of CF-GNPs as a function of time for 30 days. (k) Photographs of P-GNPs and CF-GNPs dispersed in distilled water after 24 h.

Fig. 5(a)–(c) illustrates the UV-vis spectra of water-based CF-GNPs nanofluids prepared at varied weight concentrations (0.02, 0.05, 0.08, and 0.1) wt%. A single peak was noted within wavelength ranging between 270 and 290 nm. The sharp absorption peak observed at ∼275 nm is associated with π → π * transition of the C=C bond of GNPs.^42^ Increased peak intensity due to increment in particle concentration adheres to the Beer–Lambert law.^43^

The sediment generated in the nanofluids was insignificant for particle concentrations after 30 days, which resulted in 91, 92, 92.8, and 94% with 0.02, 0.05, 0.08, and 0.1 wt% of CF-GNPs, respectively. The most suitable factors that generate high stability aqueous GNPs dispersion were determined, in terms of surfactant types (SDS, CTAB, Tween-80, and Triton X-100), and various ultrasonication probe times (15, 30, 60, and 90 min) with varied weight concentrations utilised to disperse GNPs in distilled water.^43^ The P-GNPs and varied NCF-GNPs dispersed in distilled water by UV-vis spectrum are displayed in Fig. 5(d)–(i). All the samples were placed in the wavelength that ranged from 270 to 290 nm, the peak absorption was clearly due to the presence of GNPs. A decrease in absorbance was noted for all samples, as shown in Fig. 5.^42^ Based on Fig. 5(a), the P-GNPs dispersion cannot continue in height stability for the range of ultrasonication times assessed, wherein NCF-GNPs and CF-GNPs functionalization are vital to obtaining high stability GNPs nanofluid dispersions. Hence, a 60 min ultrasonication probe time emerged as the most effective time to prepare nanofluids in this study. This 60 min ultrasonication probe time exhibited higher stability for Tw-80-GNPs sample than the other three samples for a similar amount of days, as illustrated in Fig. 5(f)–(i). Fig. 5(e) displays that SDS-GNPs at the ratio of (1 : 1) gave better stability, when compared to other tested ratios. Fig. 5(j) shows the long-term stability of CF-GNPs *via* UV-vis spectroscopy and the quantitative analysis of the dispersion state for varied weight concentrations. Over time, the CF-GNPs decreased insignificantly with relative to concentration. As a result, the maximum weight concentration of 0.1 wt% and the maximum sediment of approximately 6% gave the appropriate dispersibility of CF-GNPs. In contrast, the CF-GNPs show remarkable colloidal stability in distilled water after 24 h, as shown in Fig. 5(k).

**Fig. 6:**
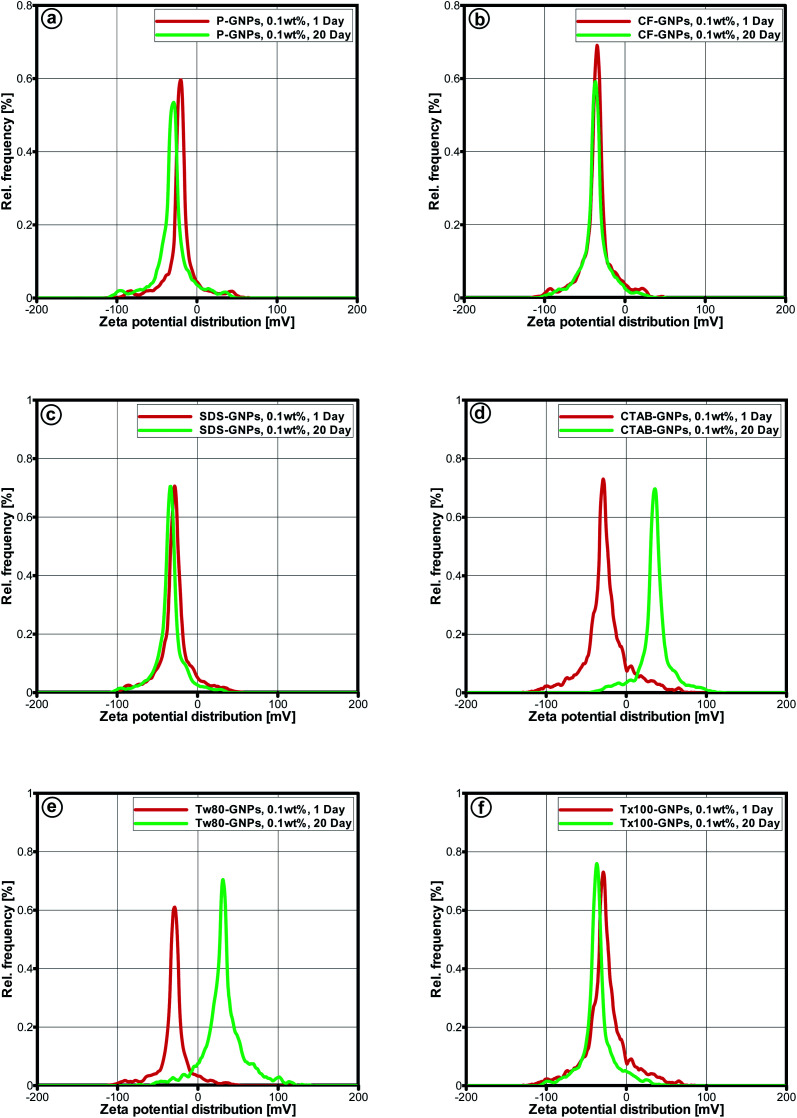
Zeta potential distribution for 1–20 days: (a) P-GNPs, (b) CF- GNPs, and (c–f) different surfactant-GNPs for the samples with particle concentration 0.1 wt%.

**Fig. 7:**
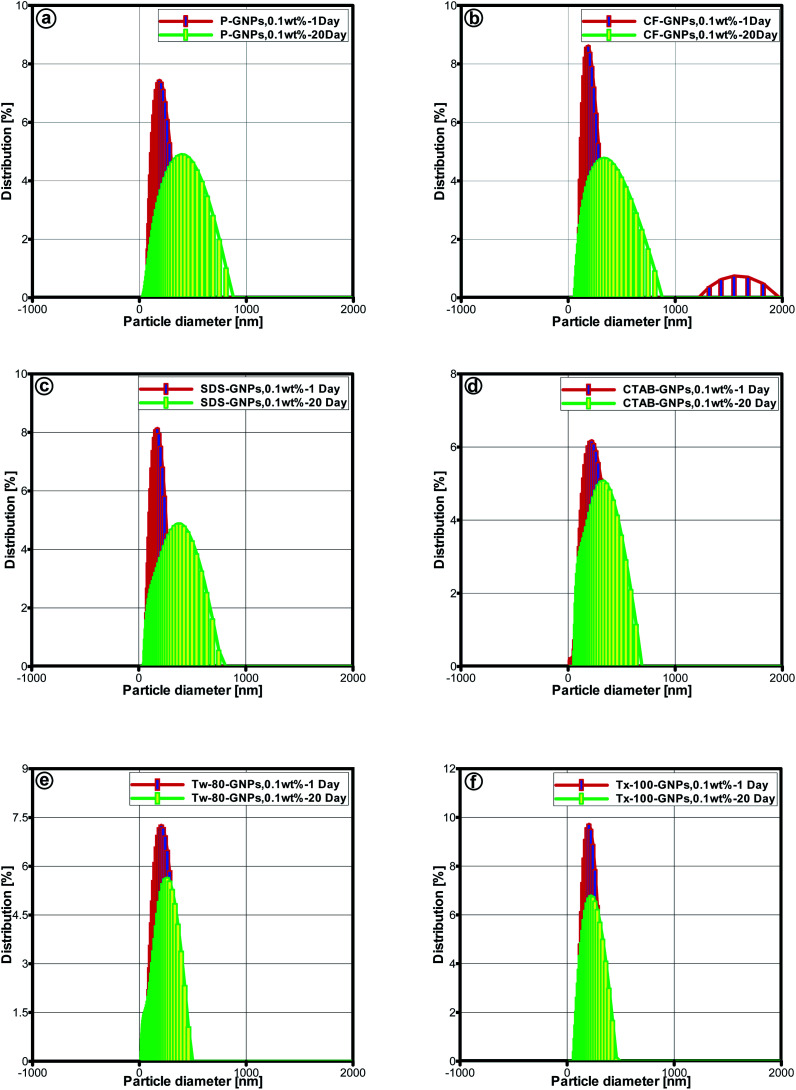
Particle size distributions for 1–20 days on (a) P-GNPs, (b) CF-GNPs and (c–f) different surfactant-GNPs particle concentration is 0.1 wt% water-based nanofluids.

Another evaluation, known as zeta potential, was applied to assess sample stability.^43^ Zeta potential can be related in a direct manner with the dispersed nanofluid stability.^44,45^ The rising repulsion between particles with similar charges determined dispersion stability. Particles with high-surface charges can lead to the low occurrence of agglomeration.^46^ Values of zeta potential for the nanofluids ranging between <−30 mV and >+30 mV were linked with physically-stable colloids.^47^Fig. 6 and 7 illustrate the values of zeta potential and particle size distribution, for both pristine GNPs and CF-GNPs samples after one and twenty days. Tables 5 and 6 tabulate the retrieved outcomes. After 20 days, CF-GNPs had a high value (−37.1 mV) and slightly minimized value of zeta potential (−35.1 mV). The pristine GNPs portrayed shallow values of zeta potential at −21.7 mV and −18.4 mV for one and twenty days, respectively. Hence, it is clear that CF-GNPs gave exceptional stability outcomes, in comparison to pristine GNPs.

**Table 5:** Zeta potential of P-GNPs, NCF-GNPs, and CF-GNPs after (1–20 days) of preparation. Note that the particle concentration is 0.1 wt%

Days	Zeta potential (mV)					
	P-GNPs	CF-GNPs	GNPs + SDS	GNPs + CTAB	GNPs + Tw-80	GNPs + Tx-100
1	−21.7	−37.1	−34.2	−34.5	−34.7	−33.7
20	−18.4	−35.1	−31.1	32.1	−32.4	−30.1

**Table 6:** The average particle size of P-GNPs, NCF-GNPs and CF-GNPs after (1–20 days) of preparation at 0.1 wt%

Days	Particle size (nm)					
	P-GNPs	CF-GNPs	GNPs + SDS	GNPs + CTAB	GNPs + Tw-80	GNPs + Tx-100
1	228.4	184.5	159.7	183.9	185.8	204.7
20	292.5	280.7	245.7	234.1	227.1	210.6

The average particle size for pristine GNPs was more prominent than that of CF-GNPs, while the dispersion of particle size for pristine GNPs was 228.4 nm and 292.5 nm after one and twenty days, respectively. The results were higher than the CF-GNPs values, whereby a dispersion of particle size had been 184.5 nm and 280.7 nm after one and twenty days, respectively. The stability of nanofluids *via* dispersal of CF-GNP generated high stability suspension, in comparison to pristine GNPs in isolation.^48,49^

### 3.2. Foaming test

**Fig. 8:**
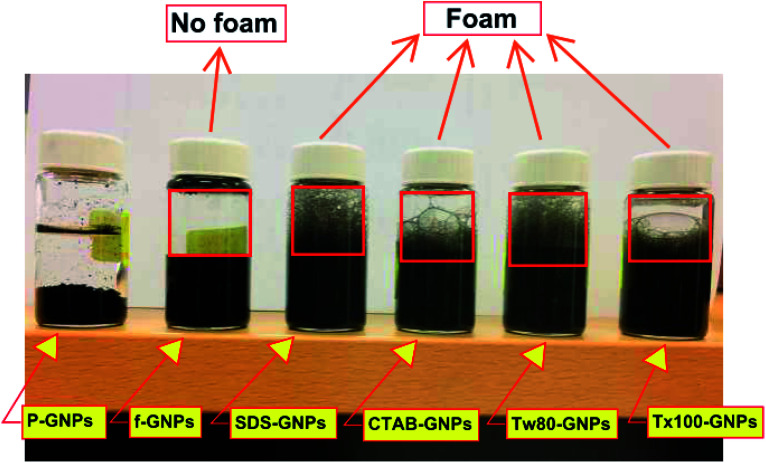
Psychical appearances of the nanofluid of samples after preparation.

Fig. 8 illustrates an image of six nanofluid samples prepared *via* shaking for 16 s to assess the generation of foam. The foam was generated above the nanofluid with the inclusion of surfactants (CTAB, SDS, Tw-80, and Tx-100), except for CF-GNPs, which did not form foam. This reflects an exceptional setting for heat transfer and fluid flow applications, as foams deteriorate the efficiency of heat transfer and interrupt fluid flow.^50^

### 3.3. Thermo-physical properties analysis

**Fig. 9:**
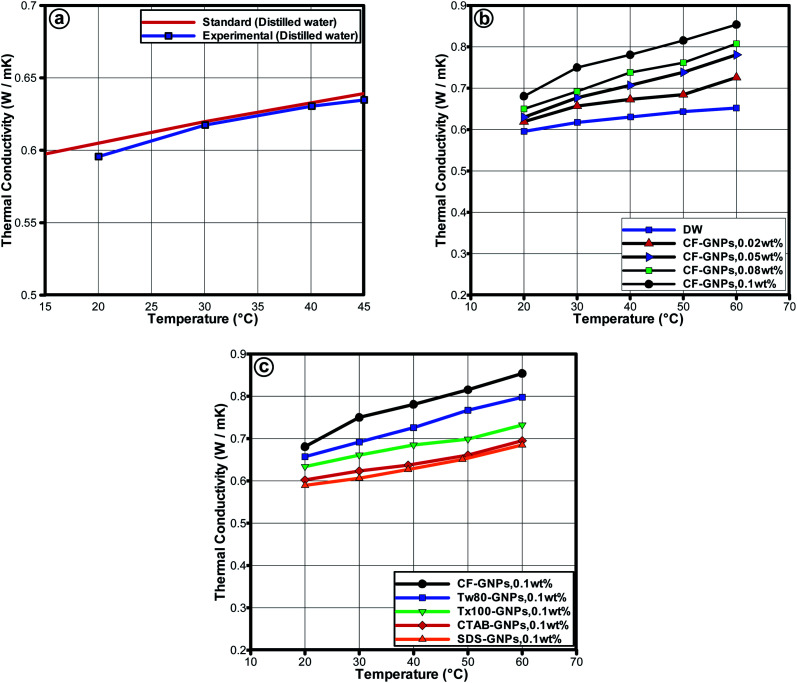
(a) Comparison of thermal conductivity values of distilled water between NIST standard^51^ and KD2 Pro thermal property analyzer measurements. (b) Thermal conductivity of CF-GNPs nanofluids as a function of temperature for different weight concentrations, (c) thermal conductivity values for CF-GNPs, and different NCF-GNPs 0.1 wt% nanofluids.

Fig. 9(a)–(c) presents the thermal conductivity of nanofluids at varying temperatures with CF-GNPs and NCF-GNPs for the following weight concentrations: (0.02, 0.05, 0.08, and 0.1) wt%. To validate the reliability of the thermal conductivity measurements for distilled water, KD2 Pro was used in the temperature range of 20–60 °C. The obtained data are in good agreement with the National Institute of Standards and Technology (NIST)^51^ as seen in Fig. 9(a). The average error in the thermal conductivity was found to be ±1.172%, indicating that the KD2 Pro thermal property analyzer is reliable to be used in the thermal conductivity measurements of the samples. Based on Fig. 9(b) and (c), the thermal conductivity of CF-GNPs nanofluids was higher than that of NCF-GNPs nanofluids and distilled water. Increment in weight concentrations of GNPs for water-based nanofluids increased its thermal conductivity. Improving thermal conductivity is a dominant function of temperature, which is attributable to Brownian motion of suspended particles.^52^ Carbon nanostructures-based nanofluid that comprises of GNPs, along with Brownian motion of GNPs and chemical treatment functionalization; the surface nanolayers tend to dominate the energy heat transfer in the nanofluids. With the presence of covalent functional groups, a higher effective heat transfer area is attributable to the higher thermal conductivity of CF-GNPs. Based on Fig. 9(b) and (c), thermal conductivity increased with increment in temperature. The agglomeration between the NPs could easily break down at higher temperatures, which may occur due to the uniform dispersion of GNPs in water. This reflects the best conduction of heat in the fluid flow, which generated layers around the GNPs of liquid molecules, thus the interface area increases the local ordering of liquid layer. It is reasonable to record higher thermal conductivity in liquid layer than bulk liquid at the interface.^53^ Increased thermal conductivity of GNPs nanofluids appears to be an essential aspect in nanolayer. The formation of layers surrounding the nanostructures on the surface of GNPs can be increased when effective heat transfer region is decreased, mainly because the non-covalent groups warp around GNPs flakes and reduce the local liquid layer by absorbing non-covalent groups at the interface area of GNPs. Oxygen-containing functional groups (COOH) on the surface of GNPs, as well as the formation of more hydrophilic phase, could lead to new homogeneous dispersion in base fluids.^53^ Hence, to enhance the aspect of dispersibility upon utilizing surfactant of carbon nanostructures, the surfactants tend to wrap around them, thus lowering micro convection that hinders the nanolayer from improvement.^54^ More recent works that compared the carbon-based nanofluids are listed in Table 7. Based on Table 7, improve thermal conductivity offers significant evidence that CF-GNPs nanofluids, in comparison to other samples, had higher concentrations of additives or NPs. It is highlighted here that medium-temperature applications can achieve suitable thermal conductivity by using low-weight GNPs concentration.

**Table 7:** Summary of experimental investigations on thermal conductivity of GNPs nanofluids

NP type	Base fluid	Particle size	Concentration (wt%)	T.C enhancement (%)	Notes	Ref.
GNPs 500	DW	Thick. 2 μm	0.1	15.87	Temperature effect	40
		Size 2 nm				
GNPs 750	DW	Thick. 2 μm	0.1	27.64	Temperature effect	56
		Size 2 nm				
GNPs 500	DW	Thick. 2 μm	0.1	25	Temperature effect	56
		Size 2 nm				
**GNPs 750**	**DW**	Thick. 2 μm	**0.1**	**29.2**	**Temperature effect**	**Present study**
		Size 2 nm				

**Fig. 10:**
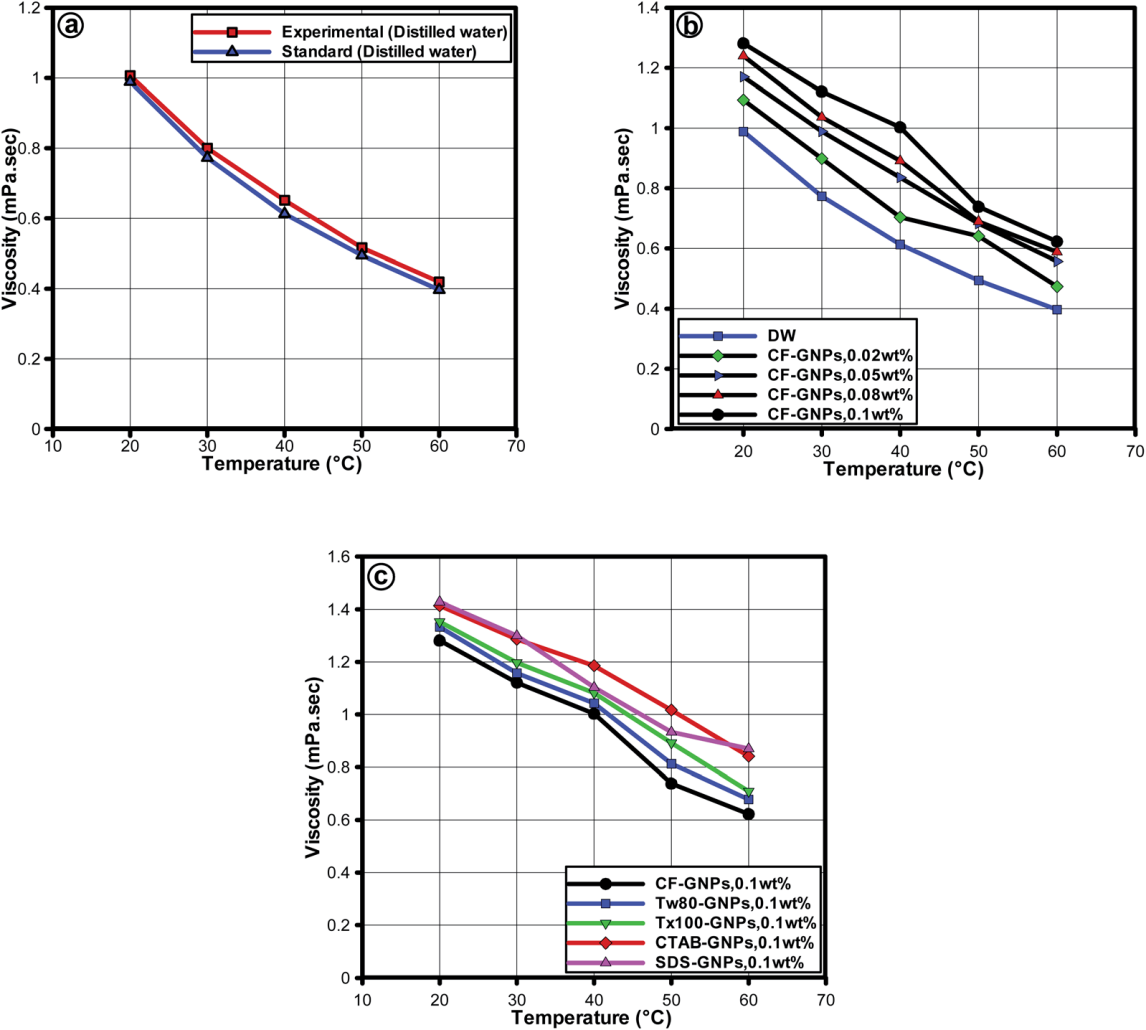
Viscosity *versus* temperature for (a) distilled water between NIST standard^51^ and experiments. Viscosity of the GNPs-based water nanofluids (b) CF-GNPs and (c) NCF-GNPs as a function of concentration and temperature at a shear rate of 200 s^−1^.

The viscosity in characterizing the suitability of nanofluids is a significant parameter for heat transfer applications. The viscosity measurements obtained using the rotational rheometer have shown good agreement with NIST standards as seen in Fig. 10(a).^51^ The average error was found to be ±3.25% indicating the reliability of the rotational rheometer to be used for measuring the viscosity of the samples. Fig. 10(b) and (c), displays the CF-GNPs nanofluids for the viscosity of distilled water as a function of weight concentration and at 20–60 °C range with fixed shear rate of 200 s^−1^. The results were obtained with increased weight concentration of nanofluids, primarily because of the increased concentration for the viscosity of nanofluids demands a direct influence on the fluid internal shear stress.^55^ Increment in temperature declined the viscosity, due to the waning of inter-molecular and inter-particle bond forces.^55^ At 0.1 w% at 60 °C, the viscosity increased by approximately 27%, when compared to the viscosity of base fluid at the same condition. As shown in Fig. 10(c), at the concentration of 0.1 wt%, the NCF-GNPs nanofluid had a higher viscosity than that of CF-GNPs nanofluid at similar concentrations, mainly due to the influence of surfactant-GNPs on the viscosity of the nanofluids. This is the main reason for the increase of viscosity in the surfactants (SDS, CTAB, Tw-80, and Tx-100) of nanofluids.

## 4. Conclusion

The current study focused on the stability behavior of functionalized GNPs water-based nanofluids for different types of surfactants (SDS, CTAB, Tw-80, and Tx-100) and different weight concentrations of (0.02, 0.05, 0.08, and, 0.1) wt%. The prepared samples were characterized using various measurement tools such as FESEM, FETEM, FTIR, Raman, TGA, EDX, XRD, UV-vis, zeta potential, and particle size distribution. The thermophysical properties were tested at a different temperature span to examine the heat transfer enhancements. The following findings would be drawn:

(1) The functionalization was confirmed by the appearance of peaks at 3410 (3000–2800) and 1650 cm^−1^ for the –OH, C–H stretching vibrations and C=O bending vibration, respectively.

(2) An increase in the *I*_D_/*I*_G_ ratio reveals that the number of sp^2^ hybridized carbons changed to sp^3^ hybridization carbons because of the covalent functionalization.

(3) The mass change of the functionalized samples with the shift in temperature observed at 24% while for pristine was only 14% for 500–800 °C.

(4) The morphology and surface deterioration of the functionalized samples were compared to the pristine to study the structure of GNPs.

(5) The sonication time showed an impact on the absorption 0.1 wt% of CF-GNPs provided higher absorption compared to the other concentrations. The ration of (1 : 1) in the non-covalent functionalization presented a higher absorption rate, among different ratios the maximum sediment of about 6%, which confirmed the appropriate dispersibility of CF-GNPs.

(6) Particle size distributions and zeta potential were the standard procedures for characterization of dispersion stability of the colloids by measuring the magnitude of electrostatic interaction between colloidal particles.

(7) To discuss the foaming test, six samples were tested and CF-GNPs were with no foam which is required for the heat transfer application to prevent the blockage inside the piping lines.

(8) The present study showed a 29.2% enhancement of the thermal conductivity at 0.1 wt%. At 0.1% weight concentration with 60 °C of nanofluid the viscosity increases of about 27% compared to the viscosity of the base fluid.

## Abbreviations

DLS: Dynamic light scattering
ELS: Electrophoretic light scattering
GNPs: Graphene nanoplatelets
MWCNTs: Multi-walled carbon nanotubes
SDS: Sodium dodecyl sulfate
SSA: Specific surface area (m^2^ g^−1^)
T.C: Thermal conductivity
UV-vis: Ultraviolet-visible spectrophotometry
DSC: Differential scanning calorimeter
FESEM: Field emission scanning electron microscopy
FTIR: Fourier transform infrared spectroscopy
P-GNPs: Pristine GNPs
FETEM: Field emission transmission electron microscopy
Tween-80: Tw-80
Triton X-100: Tx-100
SDBS: Sodium dodecyl benzene sulfonate
GA: Gum arabic
CTAB: Cetyltrimethylammonium bromide
CF-GNPs: Covalent functionalization GNPs
NCF-GNPs: Non-covalent functionalization GNPs
CNTs: Carbon nanotubes
DW: Distilled water
EG: Ethylene glycol
TGA: Thermo-gravimetric analysis
EDX: Energy dispersive spectroscopy
XRD: X-ray diffraction

## Author contributions

Conceptualization, Omar A. Hussein; Formal analysis, Omar A. Hussein; Funding acquisition, Khairul H., Ali S. Muhsan and R. Saidur; Investigation, Omar A. Hussein, Omer A. Alawi and Syed S.; Methodology, Omar A. Hussein; Project administration, Omar A. Hussein; Resources, Khairul H. and R. Saidur; Supervision, Khairul H., and Ali S. Muhsan; Writing-original draft, Omar A. Hussein; Writing-review & editing, Omer A. Alawi and Syed S.

## Funding

Universiti Teknologi PETRONAS, Khairul Habib would like to acknowledge the financial support provided by Universiti Teknologi PETRONAS through YUTP grant the project no. 015LCO-118 SUNWAY University, “R. Saidur would like to acknowledge the financial support provided by the Sunway University through the project no. STR-RCTR-RCNMET-001-2019”.

## Conflicts of interest

The authors declare no conflicts of interest.

## Supplementary Material
